# Generation of a human *Tropomyosin 1* knockout iPSC line

**DOI:** 10.1101/2023.05.03.539242

**Published:** 2023-05-04

**Authors:** Madison B Wilken, Jean Ann Maguire, Lea V Dungan, Alyssa Gagne, Catherine Osorio-Quintero, Elisa A Waxman, Stella T Chou, Paul Gadue, Deborah L French, Christopher S Thom

**Affiliations:** a Division of Neonatology, Children’s Hospital of Philadelphia, Philadelphia, PA, USA; b Center for Cellular and Molecular Therapeutics, Children’s Hospital of Philadelphia, Philadelphia, PA, USA; c Department of Pediatrics, University of Pennsylvania, Philadelphia, PA, USA; d Division of Hematology, Children’s Hospital of Philadelphia, Philadelphia, PA, USA; e Department of Pathology and Laboratory Medicine, Children’s Hospital of Philadelphia, Philadelphia, PA, USA

## Abstract

The CHOPWT17_TPM1KOc28 iPSC line was generated to interrogate the functions of *Tropomyosin 1* (*TPM1*) in primary human cell development. This line was reprogrammed from a previously published wild type control iPSC line.

## Resource Utility

The CHOPWT17_TPM1KOc28 iPSC line was generated to interrogate *Tropomyosin 1* (*TPM1*) biology (Gunning and Hardeman, 2017). The actin-regulatory *TPM1* gene impacts blood traits and developmental hematopoiesis (Thom et al., 2020). This iPSC line can facilitate study of *TPM1* in multiple developmental contexts.

## Resource Details

**Figure 1.**
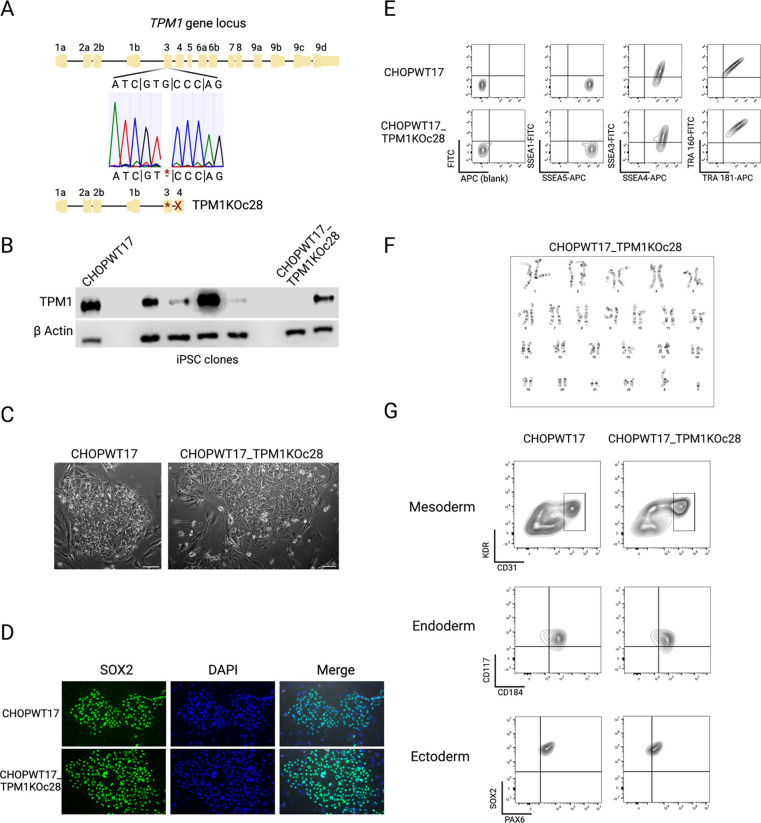
Characterization of the human iPSC line CHOPWT17_TPM1KOc28. A. Sequencing analysis of the targeted iPSC clone showing homozygous deletion at the indicated site. B. Western blot showing absence of TPM1 protein in the selected clone. C. Morphologic appearance of CHOPWT17_TPM1KOc28 iPSC clones match the parental cell line. Scale bar, 50 μm. D. Immunofluorescence staining confirms intranuclear SOX2 staining in CHOPWT17_TPM1KOc28 iPSCs and parental cells. Scale bar, 50 μm. E. Flow cytometry analysis shows appropriate stem cell marker expression in CHOPWT17_TPM1KOc28 iPSCs and parental cells. F. Representative normal karyotype for CHOPWT17_TPM1KOc28 iPSCs. G. Directed differentiation shows efficient production of mesoderm (including mesoderm-derived KDR^+^/CD31^+^ endothelial cells shown in box), endoderm, and ectoderm.

Whole blood (BioIVT, Cell Line #1000, HUMANWBNE1803609) from a 51-year-old Caucasian male was used to generate a previously reported parental CHOPWT17 iPSC line (An et al., 2022). Using CRISPR/CAS9 genome editing of CHOPWT17 iPSCs, a homozygous deletion at codon 304 (deleted C) was introduced and confirmed by DNA sequencing (Fig. 1A). This resulted in a frameshift (fs) mutation that caused a premature stop codon at position 127 (X127). Western blot confirmed *TPM1* knockout (Fig. 1B). Thus, this iPSC line abrogates *TPM1* expression in iPSCs without loss of intronic regulatory regions, which differs from previously published iPSC lines (Thom et al., 2020). This selected clone, CHOPWT17_TPM1KOc28, was then expanded, cryopreserved at passage 40, and subsequently analyzed (Tables 1 and 2). CHOPWT17_TPM1KOc28 iPSCs showed cellular morphology and intranuclear SOX2 expression identical to its wild-type parental iPSC line (Fig. 1C and 1D). Expression of stem cell surface markers was normal by flow cytometry (Fig. 1E). A normal karyotype (46, XY) was revealed by G-band analysis (Fig. 1F). Pluripotency potential was shown by differentiation into three germ layers, followed by flow cytometric analyses that confirmed relevant cell surface marker expression (Fig. 1G and Supp Fig. 1A). DNA fingerprinting by STR analysis authenticated the genetic identity of the iPSC line in relation the parental cell line (Supp Fig. 1B) and CHOPWT17_TPM1KOc28 iPSCs tested negative for Mycoplasma (Supp Fig. 1C).

## Materials and Methods

### 1. Cell Culture

The iPSCs were maintained on irradiated Mouse Embryonic Fibroblasts (MEFs) in 6-well tissue culture plates at 37°C with 5% CO_2_ and 5% O_2_ They were grown in human embryonic stem cell (hESC-10) medium made up of a base of DMEM/F12 (50:50 Gibco) with 20% knock-out serum replacement (KOSR, Gibco), 100 μM nonessential amino acids, 2 mM glutamine, 50 U/ml penicillin, 50 μg/ml streptomycin (all from Invitrogen), 10^−4^ M β–mercaptoethanol (Sigma, St. Louis, MO), and 10 ng/ml human bFGF (BioTechne). Cells were passaged every 5–7 days once they reached about 70–80% confluency using TrypLE cell dissociation reagent (Gibco) and gentle scraping before being replated onto new MEFs. Cells were transitioned in hESC-10 medium containing with 10 μM ROCK inhibitor (Y27632 dihydrochloride, Tocris) for <24 hours before replacing the media with fresh hESC-10 medium without ROCK inhibitor. Directed differentiations were performed essentially as described for mesoderm (Thom et al., 2020), endoderm (Leavens et al., 2021), and ectoderm (Telezhkin et al., 2016).

### 2. Primary cell source and CRISPR/CAS9 genome editing

The reprogrammed parental iPSC line (CHOPWT17.6) has been previously reported (An et al., 2022). Using a CRISPR/CAS9 protocol (Maguire et al., 2022), guide RNAs were designed to target exon 3 of the *TPM1* gene (GGAAGAGTTGGATCGTGCCCAGG, CCTGGGCACGATCCAACTCTTCC). The parental cell line was plated on irradiated MEFs to reach 80–85% confluency after an overnight incubation. The following quantities were used for transfection: 50 μl base medium (DMEM/F12), 0.5 μg Cas9-GFP, 0.5 μg of each gRNA and 3 μl Lipofectamine Stem Reagent. After 48 hours, FACS-sorted GFP^+^ cells were plated on a 10cm plate containing 1:3 matrigel and MEFs, then allowed to grow until colonies were established. Colonies were manually picked and expanded for screening. Targeted clones were identified using PCR-based screening strategies designed to detect editing. The chosen clone that suggested homozygous knockout was then sequenced and further analyzed to confirm *TPM1* knockout.

### 3. Karyotype analysis

Cytogenic analysis was performed on twenty G-banded metaphase cells by Cell Line Genetics, Inc (Madison, WI). All 20 cells demonstrated a normal male karyotype.

### 4. Flow cytometry

Single cells, collected using trypsin dissociation, were stained and analyzed with a CytoFLEX flow cytometer (Beckman Coulter) and FlowJo software (BD). Cells were incubated in the dark for 15 min at room temperature with the following antibodies to evaluate pluripotency expression: Alexa-Fluor^®^-647 α-human SSEA4 (1:400) and Alexa-Fluor^®^-488 SSEA3 (1:50); Tra-1–81 (1:50) and Tra-1–60 (1:50); Alexa-Fluor^®^-488 SSEA1 (CD15) (1:50) and Alexa-Fluor^®^-647 SSEA5 (1:200) (BioLegend). In all experiments, an unstained sample was used as a negative control.

### 5. Western blot

Cell pellets from selected iPSC clones were lysed in RIPA buffer (Cell Signaling Technologies) and proteins electrophoretically separated on Nu-PAGE polyacrylamide gels (Invitrogen). Proteins were transferred to nitrocellulose membranes (Invitrogen), blocked in 3% milk in TBS-Tween, and probed with antibodies to detect TPM1 (1:1000, Cell Signaling Technologies) or β-Actin (1:5000, Sigma). Secondary antibodies were anti-Rabbit IgG-HRP or anti-Mouse IgG-HRP (Bio-Rad). Western blots were imaged using a Chemi-Doc Imaging System (Bio-Rad).

### 6. Immunocytochemistry

Cells were plated in hESC-10 media on irradiated MEF-coated coverslips at 90% confluency and fixed in 4% paraformaldehyde for 15 min at room temperature. Permeabilization was performed using ice cold 100% methanol for 10 min at −20°C. Blocking and antibody buffers were prepared in 0.3% Triton-X100/PBS with 5% goat serum and Hank’s Balanced Salt Solution (HBSS), respectively. Cells were blocked at room temperature for 1 h with gentle rocking. Primary antibody (SOX2) incubation was performed at 4°C overnight, and secondary antibody (Goat α-Rabbit AF488) incubation at room temperature for 2 h. Coverslips were mounted in Vectashield mounting media with DAPI (Vector Laboratories, Burlingame, CA). Staining was visualized on an Olympus IX70 microscope and imaged with Metamorph (Molecular Devices).

### 7. STR analysis

The genetic integrity of the iPSC line was confirmed by DNA fingerprinting. STR analysis was done by Cell Line Genetics, Inc (Madison, WI).

### 8. Mycoplasma testing

Mycoplasma testing was performed by PCR amplification on iPSC genomic DNA. Cycling parameters were as follows: 95°C for 10 min, 35 cycles of 95°C 45 sec, 55°C 30 sec, 72°C 30 sec, a 72°C 10-minute extension, and a 4°C hold. PCR products were separated on a 1.0% agarose gel and visualized with ethidium bromide. Positive, and negative controls were used.

## Supplementary Material

Supplement 1

## Figures and Tables

**Table 1.** Characterization and validation

Classification	Test	Result	Data
Mutation Analysis	Sequencing	Homozygous c.304delG (p.A102PfsX127)	Fig. 1A
	Western blot	Absent TPM1	Fig. 1B
Morphology	Photography	Normal	Fig. 1C
Phenotype	Immunocytochemistry	Staining of marker SOX2	Fig. 1D
	Flow cytometry	SEA3/4, Tra 1–60/81	Fig. 1E
Genotype	Karyotype (G-Banding) and resolution	Normal, XY; Resolution: fair	Fig. 1F
Differentiation potential	*In vitro* differentiation	FACS staining for KDR/CD31 (mesoderm/endothelium), CD117/CD184 (endoderm), or SOX2/PAX6 (ectoderm). FOXG1 also confirmed ectoderm identity	Fig. 1G and Supp Fig 1A
Identity	STR Analysis	Matched iPSCs to parental line	Supp Fig. 1B
Microbiology and virology	Mycoplasma	Negative by PCR	Supp. Fig. 1C
Donor screening	HIV 1 + 2, Hepatitis B, Hepatitis C	N/A	N/A
Genotype additional info	Blood group genotyping	N/A	N/A
	HLA tissue typing	N/A	N/A

**Table 2:** Reagent details

	**Antibody**	**Dilution**	**Company Cat # and RRID**
Pluripotency Markers (Flow Cytometry)	SSEA-1	1:50	BioLegend, Cat# 301910, RRID: AB_493257
	SSEA-3	1:50	BioLegend, Cat# 330306, RRID: AB_1279440
	SSEA-4	1:400	BioLegend, Cat# 330408, RRID: AB_1089200
	SSEA-5	1:200	BioLegend, Cat# 355210, RRID: AB_2562013
	Tra-1–60	1:50	BioLegend, Cat# 330614, RRID: AB_2119064
	Tra-1–81	1:50	BioLegend, Cat# 330706, RRID: AB_1089242
Sternness Markers (ICC and Flow Cytometry)	SOX2	1:300	Cell Signaling, Cat# 3579S, RRID: AB_2195767
Differentiation Markers (flow cytometry)	KDR for mesoderm	1:20	R&D, Cat# FAB357P, RRID: AB_357165
	CD31 for mesoderm-derived endothelium	1:100	BioLegend Cat# 303118, RRID: AB_2247932
	CXCR4 (CD184) for endoderm	1:100	BD Pharmigen, Cat#560936, RRID: AB_398616
	C-Kit (CD117) for endoderm	1:100	BioLegend, Cat# 313204, RRID: AB_314983
	FOXG1 for ectoderm	1:300	Abcam, Catalog #ab196868, RRID: AB_2892604
	PAX6 for ectoderm	1:20	BD, Catalog #562249, RRID: AB_1115295
Western Blot	TPM1 (TPM1.6/1.7 isoforms)	1:1000	Cell Signaling Technology Cat# 3910, RRID: AB_2205654
	β-Actin	1:5000	Sigma-Aldrich Cat# A1978, RRID: AB_476692
Secondary Antibodies (flow cytometry)	Goat anti Rabbit IgG (H + L) AF488	1:400	Jackson ImmunoResearch, Cat# 111–545-144, RRID: AB_2338052
Secondary Antibodies (western blot)	Goat anti-Rabbit IgG-HRP conjugate	1:5000	Bio-Rad Cat# 1706515, RRID: AB_2617112
	Goat anti-Mouse IgG-HRP conjugate	1:5000	Bio-Rad Cat# 1706516, RRID: AB_2921252

**Primers**			
	**Target**	**Forward/Reverse Primer (5’–3’)**	
Targeted mutation creation and analysis/sequencing	TPM-1 STOP gRNA 5F	GGAAGAGTTGGATCGTGCCCAGG	
	TPM-1 STOP gRNA 5R	CCTGGGCACGATCCAACTCTTCC	
	TPM-1 4F	GATGAGGGCTTGTAATGCAC	
	TPM-1 4R	CTGTCTCCCTCATTAGATGGTG	
Mycoplasma Detection (PCR)	Myco-F	CGCCTGAGTAGTACGTTCGC	
	Myco-R	GCGGTGTGTACAAGACCCGA	

**Resource Table:** 

Unique stem cell line identifier	CHOPi013-A
Alternate name of stem cell line	CHOPWT17_TPM1KOc28
Institution	The Children’s Hospital of Philadelphia (CHOP), Philadelphia, Pennsylvania, USA
Contact information of distributor	Christopher S. Thom, thomc@chop.edu
Type of cell line	iPSC
Origin	Human
Additional origin info	Age: 51 years Sex: Male Ethnicity: Caucasian Type: O−
Cell source	Blood
Clonality	Clonal
Method of reprogramming	N/A
Genetic modification	CRISPR-Cas9
Associated disease	N/A
Gene/Locus	TPM1
Method of modification	N/A
Name of transgene or resistance	N/A
Inducible/constitutive system	N/A
Date archived/stock date	February 2022
Cell line repository/bank	https://hpscreg.eu/cell-line/CHOPi013-A
Ethical approval	Source cells purchased from BiolVT (Cell Line #1000, HUMANWBNE1803609)
