# Supplementary material for: Generation of a human Tropomyosin 1 knockout iPSC line
Source: bioRxiv. 2023 May 4:2023.05.03.539242. Preprint. [Version 1] doi: 10.1101/2023.05.03.539242 (PMC10187204; doi:10.1101/2023.05.03.539242)
Supplement: Supplement 1 [file NIHPP2023.05.03.539242v1-supplement-1.pdf]

bioRxiv preprint doi: https://doi.org/10.1101/2023.05.03.539242; this version posted May 4, 2023. The copyright holder for this preprint (which was not certified by peer review) is the author/funder, who has granted bioRxiv a license to display the preprint in perpetuity. It is made available under aCC-BY 4.0 International license.

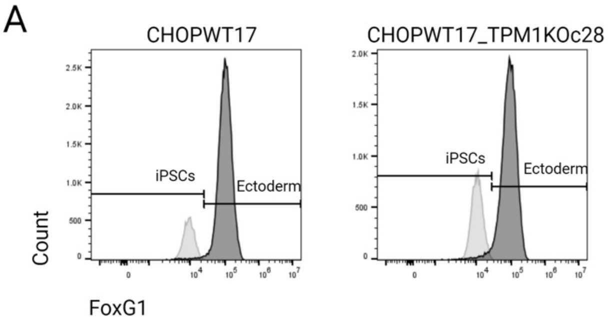

B

Amelogenin	X	Y	D18S51	13	14	D5S818	12	
D3S1358	14	18	D2S1338	17	20	TPOX	8	12
D1S1656	12	16	CSF1PO	10		DYS391	12	
D2S441	10	14	Penta D	10	11	D8S1179	12	13
D10S1248	14	15	THO1	7	9.3	D12S391	17	24
D13S317	8	13	vWA	15	18	D19S433	14	15
Penta E	7	21	D21S11	29	31	FGA	22	25
D16S539	12		D7S820	8	9	D22S1045	14	15

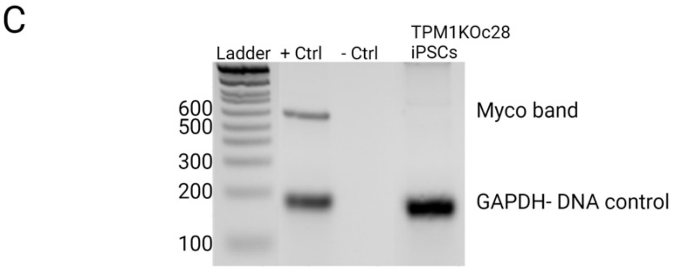

Supplementary Figure 1.

A. Directed differentiation shows efficient production of FoxG1+ ectoderm.
B. STR analysis results for CHOPWT17_TPM1KOc28 matched the parental control line.
C. Mycoplasma testing was negative for CHOPWT17_TPM1KOc28 iPSCs.
